# Malaria prevalence and mosquito net coverage in Oromia and SNNPR regions of Ethiopia

**DOI:** 10.1186/1471-2458-8-321

**Published:** 2008-09-21

**Authors:** Estifanos B Shargie, Teshome Gebre, Jeremiah Ngondi, Patricia M Graves, Aryc W Mosher, Paul M Emerson, Yeshewamebrat Ejigsemahu, Tekola Endeshaw, Dereje Olana, Asrat WeldeMeskel, Admas Teferra, Zerihun Tadesse, Abate Tilahun, Gedeon Yohannes, Frank O Richards

**Affiliations:** 1 The Carter Center, Addis Ababa, Ethiopia; 2 University of Cambridge, Department of Public Health and Primary Care, Cambridge, UK; 3 The Carter Center, Atlanta, Georgia, USA; 4 Oromia Regional Health Bureau, Addis Ababa, Ethiopia; 5 Southern Nations, Nationalities and Peoples' Regional Health Bureau, Awassa, Ethiopia; 6 Disease Prevention and Control Department, Ministry of Health, Addis Ababa, Ethiopia

## Abstract

**Background:**

Malaria transmission in Ethiopia is unstable and seasonal, with the majority of the country's population living in malaria-prone areas. Results from DHS 2005 indicate that the coverage of key malaria interventions was low. The government of Ethiopia has set the national goal of full population coverage with a mean of 2 long-lasting insecticidal nets (LLINs) per household through distribution of about 20 million LLIN by the end of 2007. The aim of this study was to generate baseline information on malaria parasite prevalence and coverage of key malaria control interventions in Oromia and SNNPR and to relate the prevalence survey findings to routine surveillance data just before further mass distribution of LLINs.

**Methods:**

A 64 cluster malaria survey was conducted in January 2007 using a multi-stage cluster random sampling design. Using Malaria Indicator Survey Household Questionnaire modified for the local conditions as well as peripheral blood microscopy and rapid diagnostic tests, the survey assessed net ownership and use and malaria parasite prevalence in Oromia and SNNPR regions of Ethiopia. Routine surveillance data on malaria for the survey time period was obtained for comparison with prevalence survey results.

**Results:**

Overall, 47.5% (95% confidence interval (CI) 33.5–61.9%) of households had at least one net, and 35.1% (95% CI 23.1–49.4%) had at least one LLIN. There was no difference in net ownership or net utilization between the regions. Malaria parasite prevalence was 2.4% (95% CI 1.6–3.5%) overall, but differed markedly between the two regions: Oromia, 0.9% (95% CI 0.5–1.6); SNNPR, 5.4% (95% CI 3.4–8.5), p < 0.001. This difference between the two regions was also reflected in the routine surveillance data.

**Conclusion:**

Household net ownership exhibited nearly ten-fold increase compared to the results of Demographic and Health Survey 2005 when fewer than 5% of households in these two regions owned any nets. The results of the survey as well as the routine surveillance data demonstrated that malaria continues to be a significant public health challenge in these regions–and more prevalent in SNNPR than in Oromia.

## Background

Despite a control program lasting over 40 years, the majority of Ethiopia's population is still at risk from malaria. In most of the country, transmission is unstable and seasonal, with occasional devastating epidemics [1,2]. Malaria is the most frequent cause of out-patient presentation and in-patient admission nationwide, and is second only to respiratory tract infections as a cause of death in children [3]. The people of Ethiopia also suffer from many of the 'neglected tropical diseases' including river blindness (onchocerciasis) [4] and trachoma [5].

There is relatively little information from representative surveys on the distribution of malaria in Ethiopia in a typical year or on the coverage of malaria control interventions. This study was designed to partially fill that gap and describes the results of a household survey done in Oromia and SNNPR regions in January 2007. In addition, the Ethiopian Ministry of Health (MOH) collects a large amount of routine data on malaria, and we wished to determine the usefulness of this information for assessing the effectiveness of malaria control in these two regions. Furthermore, we sought to compare the results with that of Ethiopia Health and Demographic Survey (EDHS 2005) that was conducted from April-August 2005. EDHS 2005 indicated that the coverage of major malaria control interventions was low.

The Carter Center (TCC) has been assisting the Ethiopian MOH with onchocerciasis control for eight years, in collaboration with the African Programme for Onchocerciasis Control (APOC), the Lions SightFirst Program and other Non Governmental Organizations (NGOs) [6]. Over 2.5 million people have been treated annually since 2001 with ivermectin (Mectizan^®^, donated by Merck & Co. Inc.) through the community-directed treatment with ivermectin (CDTI) program in areas of Oromia and SNNPR regions assisted by Lions-Carter Center.

At the request of the Ethiopian government in early 2006, The Carter Center became a partner in the malaria program with a focus on helping to reach the national goal of full population coverage of long-lasting insecticidal nets (LLINs), defined as a mean of 2 LLIN per household. The plan required distribution of about 20 million LLIN by the end of 2007, free of charge to a population of approximately 50 million people of all ages. The Carter Center assisted in distribution of 3 million LLIN in the regions of Amhara, Oromia and SNNPR.

Long-lasting insecticidal nets are being distributed in all malarious areas of Ethiopia with the assistance of health extension workers, volunteer community workers and local administration. However, even if all households at risk are fully covered, nets must also be used consistently and correctly if they are to have maximum impact. At present over 30,000 ivermectin community drug distributors (CDDs) are working in the TCC-assisted onchocerciasis control program areas – approximately one per 30 to 50 households. We plan to investigate the effectiveness of involving CDDs in promotion of LLIN use and ensuring full net coverage. Another TCC program focussed on trachoma was similarly redesigned to integrate with malaria control efforts of the Amhara regional government [7].

## Methods

### Study sites

**Figure 1:**
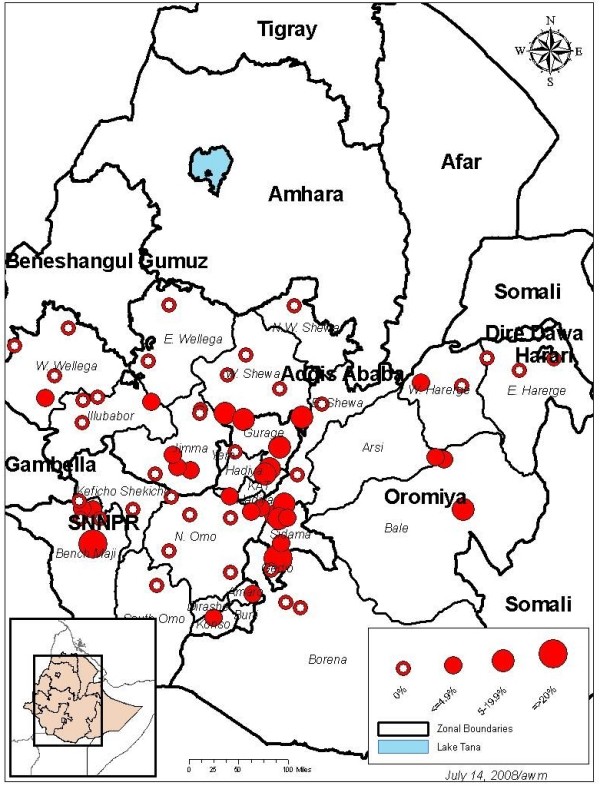
Map of Oromia and SNNPR Regions showing malaria parasite prevalence in January 2007.

The survey was conducted in Oromia and SNNPR regions in January 2007. Oromia is located in south-central and western Ethiopia and has an estimated population of 26,553,000; whereas SNNPR Region lies in the south-west and has an estimated population of 14,901,990 (Figure 1) [8]. Since 2001, The Carter Center-assisted onchocerciasis control program has been implementing CDTI in these two regions where 54% of the country's population live. The regions are divided into administrative units which include, in descending order: zone, woreda, kebele and stateteam. The stateteam, also called Got/Garre in some areas, is the lowest administrative unit and comprises an average of 50 households or about 250 people with an elected representative.

The two regions (Oromia and SNNPR) were the main domains for the survey. Two other overlapping domains within the regions were defined based on the presence of onchocerciasis and thus coverage by the Carter Center CDTI program: CDTI areas and non-CDTI areas, with the aim that baseline net utilization would be estimated in these areas for future comparison. Our hypothesis is that the involvement of CDDs in LLIN promotion will improve LLIN utilization in CDTI areas. Only TCC-assisted onchocerciasis areas were intensively assessed; two other zones where CDTI is managed by another organization (East and West Wellega) were included in the survey but not over-sampled. Kebeles known to be non-malarious were excluded from the sampling frame.

### Sample size estimation

The sample was estimated to determine prevalence of malaria and mosquito net coverage and use within: 1) each of the two regions; 2) the CDTI areas of the two regions combined; and 3) non-CDTI areas of the two regions combined. We calculated our sample size for malaria parasite prevalence testing assuming an expected prevalence of 8.0%, 2.0% margin of error for 95% confidence interval (CI), 5% level of significance, design effect of 1.2, and up to 20% allowance for non-response. A sample of 1,000 people (~200 households) was estimated as the minimum number required for malaria parasite prevalence testing per domain.

For estimating sample size for net use, we assumed 25% of children under-five years old slept under a net the previous night, with 5% margin of error for 95% CI, 5% level of significance, and design effect of 1.2, meaning we would need 381 children under-five years in each domain, which we would expect to find in ~400 households (16 clusters of 25 households). We decided to have 16 clusters (400 households) in our smallest domain (CDTI areas) to assess mosquito net coverage, but to take blood slides from alternate households (200 households per domain).

For logistical reasons, each region was stratified into four quadrants, in each of which eight clusters were selected. The CDTI areas comprised 2 quadrants (one in each region with a total of 16 clusters) while the remaining six quadrants (48 clusters) formed the non-CDTI domain. This gave 32 clusters per region and a total of 64 clusters. Comparison of the results between TCC-assisted CDTI areas and non-CDTI areas will be reported separately.

### Sample selection

To select 200 households in each quadrant, we used a multi-stage cluster random sampling design. In each quadrant, eight kebeles (clusters) were selected using probability proportional to population size. Within the kebele, five stateteams (which are all approximately similar in size) were selected by lottery, literally drawing the names out of a hat at the kebele office. In the final stage, five households were selected from the state team using the random walk method [9]. All members of the selected households were included in the sample.

### Household questionnaire

The survey questionnaire was based on the Malaria Indicator Survey Household Questionnaire, modified for the local conditions [10]. The questionnaire was translated and printed in Amharic and Oromiffa languages and field-tested in non-survey kebeles to determine the validity of the pre-coded answers. Interviews were conducted with the head of household, or another adult if the head of household was absent or unable to respond for any reason. If interviews were conducted with someone other than the head of household then the respondent was requested to answer as though he or she were the head of household. The data collection form had two parts: household questionnaire and malaria parasite prevalence.

In the household questionnaire, respondents were asked about: proxy indicators of wealth (electricity in the household, possession of a functioning radio set, and possession of a functioning television set); room construction materials; indoor residual spraying; presence and type of mosquito net (verified by observation); demographic information on residents; and where people slept. Interviewers asked to see each net by room in the house, determined whether it was a LLIN or not, and asked who slept under it the previous night. The questionnaire was also designed to determine whether any household residents slept outside, and if so whether they used a net (ITN, LLIN or untreated) or LLIN.

### Malaria parasite prevalence

Consenting residents of even-numbered households were recruited for the malaria parasite prevalence survey. Participants had both a rapid diagnostic test (RDT), which gave an on-the-spot diagnosis, and provided thick and thin blood films for microscopy. The rapid diagnostic test used was ParaScreen (Zephyr Biomedical Systems, ) which detects both P. falciparum and other plasmodia species (most likely P. vivax in the Ethiopian context). The test uses approximately 5 μl of blood and is readable after 15 minutes. Participants with positive rapid tests were offered treatment according to national guidelines: CoArtem^® ^for P. falciparum infection, chloroquine for other malaria infection, and clinic-based quinine therapy for self-reported pregnant women [11].

While the RDTs were used to provide rapid treatment for infected persons in the field, the parasite prevalence survey was based on microscopic examination of stained blood films. Two blood slides, each composed of thick and thin films, were taken from each participant by a medical laboratory technician according to standard WHO-approved protocol [12]. Slides were labelled and air-dried horizontally in a slide tray in the field, and thin films were fixed with methanol immediately after drying. Slides were stained with 3% Giemsa for 30 minutes at the nearest health facility when the team returned from the field. Usually, field teams returned to the clinic each evening but when working in more remote areas, they were sometimes obliged to sleep in the field and stain the slides the following day. To ensure maximum participation, households with absentees were revisited on the same day to recruit those missing at the first visit. Repeat visits to recruit absentees on other days were not logistically possible.

Blood slides were read at a reference laboratory in Addis Ababa and classified qualitatively as either negative, P. falciparum positive, P. vivax positive, or mixed infection. One hundred high power fields of the thick film were examined at a magnification of 1000×, before identifying a slide as negative or positive. If positive, the thin film was read to determine the species. Parasite density was not quantified. To ensure accuracy, all positive slides and a random sample of 5% of the negative slides were re-examined by a separate microscopist, who was blinded to the diagnosis of the first slide-reader. The overall agreement between the two microscopists was 99.4%. The second slide from each participant was used if the first was broken or unreadable. The identity of survey participants who had positive blood slides but had negative RDT results was sent back to the field teams for follow-up and appropriate treatment, where necessary. Comparison of the results obtained with RDT and slides has been reported elsewhere [13].

### Quality control, data entry and analysis

Forms were checked by the supervisor in the field and inconsistencies verified with the respondents. Data were double entered by different entry clerks and compared for consistency using Census and Survey Processing System (U.S. Census Bureau Washington DC, USA). Statistical analysis was conducted using Stata™ 9.2 (Stata Corporation, College Station, Texas, USA). Sampling probabilities were calculated for kebeles, and sampling weights derived as the inverse of the product of sampling probabilities at the kebele level. Descriptive statistics were used to examine the characteristics of the sample. Differences in proportions between the regions were compared using chi-square test while t-test was used to compare means. Point estimates and confidence intervals were derived using the SURVEY (SVY) routine in Stata which controlled for clustering in the sample design as well as weighting for sampling probability [14].

### Routine surveillance data

Three-year data on reported monthly clinical cases of malaria were obtained from the integrated disease surveillance (IDS) system from July 2004 to June 2007 (Ethiopia malaria statistics are reported by the MOH fiscal year July-June). In order to assess whether the survey was done in a representative year for malaria transmission (i.e. not epidemic or abnormally low transmission) we extracted the total annual numbers of cases from the IDS data for Oromia and SNNPR regions and expressed these as incidence/1000/year.

### Ethical considerations

The protocol received ethical approval from the Emory University Institutional Review Board (IRB 1816) and the Oromia and SNNPR Regional Health Bureaus. Informed consent was sought in accordance with the tenets of the declaration of Helsinki. Verbal informed consent to participate in interviews was sought from the heads of the household. Signed informed consent for blood testing was sought from each individual and parents of children aged 17 years and younger. Personal identifiers were removed from the data set before analyses were undertaken.

## Results

### Household survey results

Survey results are reported for the overall sample and by two domains: for Oromia and SNNPR, whereas comparison of CDTI/non-CDTI areas will be reported elsewhere.

#### Characteristics of study households and participants

As shown in Table 1, a total of 1,607 households were selected for the survey. The overall mean household size was 5.5 persons (95% CI 5.3–5.8) with household size ranging from 1 to 18. Our proxy indicators of wealth (electricity, radio, TV) were reported in 3.5% (95% CI 1.2–9.6), 38.6% (95% CI 31.2–46.5), and 1.2% (95% CI 0.6–2.4) of households, respectively. The majority of households, 69.8% (95% CI 60.0–78.1), had a thatch roof; walls made from sticks, 95.4% (95% CI 89.5–98.1); and floors of compacted earth, 88.5% (95% CI 80.4–93.5).

**Table 1:** Characteristics of study households and participants

			Household characteristics				Participant characteristics		
				
Domain	Estimated population*	Clusters sampled	Number sampled	Mean size	Thatch roof (%)	Insecticide sprayed in the last year (%)	Number included in the sample	Mean age	Male (%)
Region									
Oromia	26,553,000	32	809	5.4	67.0	18.6	4,428	19.3	49.3
SNNPR	14,901,990	32	798	5.7	75.1	18.3	4,397	19.7	50.1

TOTAL	41,454,990	64	1607	5.6	69.8	18.5	8,825	19.6	49.7

* Central Statistical Agency of Ethiopia: Population July 2006

**Figure 2:**
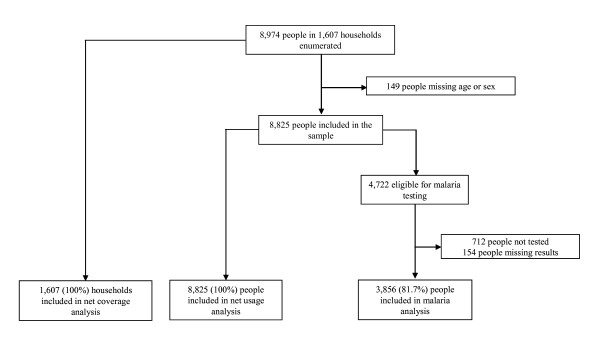
The sample population for the malaria prevalence and net coverage survey.

Figure 2 shows the sample population and those recruited for net usage interview and malaria parasite prevalence. A total of 8,974 people were enumerated of whom 149 (1.7%) were excluded from analysis due to missing data on age or sex. Net usage was assessed for all the 8,825 people included in the sample (Figure 2). Tables 2 and 3 show the household and individual characteristics of net ownership and use, respectively.

**Table 2:** Household net ownership

Domain	Any net		LLIN	
		
	% of HH with > = 1 (95% CI)	Mean (95% CI)	% of HH with > = 1 (95% CI)	Mean (95% CI)
Region				
Oromia	45.4 (27.1–65.1)	0.69 (0.37–1.02)	32.5 (17.4–52.3)	0.47 (0.20–0.74)
SNNPR	51.2 (32.7–69.3)	0.74 (0.43–1.04)	40.1 (23.8–58.9)	0.59 (0.30–0.88)

TOTAL	47.5 (33.5–61.9)	0.7 (0.5–0.9)	35.1 (23.1–49.4)	0.5 (0.3–0.7)

**Table 3:** Net usage: proportion of people who reported sleeping under a net last night

Domain	Number of people (N)			Slept under any net last night (%)			Slept under LLIN last night (%)		
			
	All people	Under fives	Pregnant women	All people	Under fives	Pregnant women	All people	Under fives	Pregnant women
Region									
Oromia	4,428	830	87	35.8	39.5	41.0	23.5	24.6	29.2
SNNPR	4,397	628	87	34.7	41.5	46.4	28.3	31.9	36.9

TOTAL	8825	1458	174	35.4	40.1	42.9	25.2	26.7	32.0

Of the 8,825 people included in the sample, the overall mean age was 19.4 years (95% CI 18.9–20.0) and 49.9% were male. There were 1458 children under five years of age (16.5% of sample) and 174 women who reported being pregnant (2.0%) living in the selected households (Table 3). The proportion of children less than five years old in the sample was higher in Oromia (18.7%) than in SNNPR (14.3%), p < 0.0001, whereas the number of pregnant women was no different between regions.

A total of 4,722 people in even numbered households were eligible for malaria testing of whom 3,856 (81.7%) were included in the analysis (Table 4). Participation was significantly higher in Oromia (85.0%) than in SNNPR (78.4%), p < 0.0001. Of the 3,856 people examined for malaria parasites, 712 (18.5%) were children under five years. The proportion of children under five in the sample tested was greater in Oromia (21.9%) than in SNNPR (14.8%), p < 0.0001.

**Table 4:** Prevalence of malaria by blood slide microscopy

Domain	Number examined	Malaria parasite prevalence (%)				Pf:Pv ratio
			
		P. falciparum only	P.vivax only	Mixed Pf and Pv	Total* % (95% CI)	
Region						
Oromia	1,996	0.7	0.1	0	0.9 (0.5–1.6)**	4.3
SNNPR	1,860	3.6	1.8	0	5.4 (3.4–8.5)**	2.1

TOTAL	3,856	1.7	0.7	0	2.4 (1.6–3.5)	2.3

CI, confidence interval; Pf, Plasmodium falciparum; Pv, Plasmodium vivax

*Total parasite prevalence does not equal the sum of the species prevalence in some rows due to rounding

** Statistically significant difference between the regions

#### Indoor residual spraying

Indoor residual spraying (IRS) in the last 12 months was reported in 18.5% (95% CI 10.2–31.1) of households (Table 1). There was no significant difference between regions in the proportion of households sprayed in the last year (18.6% Oromia vs. 18.3% SNNPR). IRS coverage varied by cluster from 0 to full coverage; in 43 of the 64 clusters (67%), no household reported IRS in the last 12 months.

#### Household net ownership

Table 2 shows that overall, at least one mosquito net (any type) was reported and verified by observation in 47.5% (95% CI 33.5–61.9) of households, with a range of 0 to 5 nets observed. Of all the nets seen in all the households, 70.1% were LLINs, and 35.1% (95% CI 23.1–49.4) of households had at least one LLIN. The overall mean number of LLINs per household was 0.5 (95% CI 0.3–0.7). There were no statistically significant differences in the proportions of households with any net or LLIN, or the mean number of any net or LLIN per household between Oromia and SNNPR regions. Household net ownership by cluster varied from 0 to 100%, with 25% of the 64 clusters having no net and 39% having no LLIN.

#### Net usage by participants

Reported net usage among the study participants is summarised in Table 3. The overall proportion of people reporting sleeping under any mosquito net the previous night was 35.4% (95% CI 24.4–48.1). Among the population with particular vulnerability to malaria, the under five year-olds and pregnant women, sleeping under a mosquito net was reported for 40.1% (95% CI 27.3–54.4) and 42.9% (95% CI 30.0–57.0), respectively. Persons reporting sleeping under an LLIN the previous night were: 25.2% (95% CI 16.3–36.9) overall; 26.7% (95% CI 17.1–39.2) of children under five years; and 32.0% (95% CI 20.6–45.9) of pregnant women. There were no differences in the proportions of people (overall, under fives and pregnant women) sleeping under any net or LLIN between Oromia and SNNPR regions. Net usage by cluster varied from 0 to 100%, with 27% of the 64 clusters having no one sleeping under a net the previous night and 41% having no one sleeping under a LLIN the previous night.

#### Malaria prevalence

The malaria parasite prevalence by blood slide microscopy is shown in Table 4. The map in Figure 1 shows prevalence and location of sampled clusters. A total of 3,856 blood slides were examined with good concordance between first and second reading (overall agreement was 99.4%). The overall malaria parasite prevalence was 2.4% (95% CI 1.6–3.5). Prevalence by cluster varied from 0 to 25%, with 55% of the 64 clusters having no positive cases.

The malaria parasite prevalence differed markedly between Oromia, 0.9% (95% CI 0.5–1.6) and SNNPR, 5.4% (95% CI 3.4–8.5) regions (p < 0.001). The prevalence was highest in the Eastern and North-eastern zones of SNNPR (Fig 1). The malaria species seen most frequently was P. falciparum: 69.4% of positive slides had P. falciparum and 30.6% had P.vivax. No mixed infections of P. falciparum and P. vivax were observed. The overall ratio of P. falciparum to P. vivax was 2.3; 4.3 in Oromia and 2.1 in SNNPR.

#### Routine surveillance data on malaria

For the data obtained from the IDS system, completeness of reporting during July 2006 – March 2007 (which included the survey period in January 2007) was 77% in Oromia and 92% in SNNPR. Reporting completeness here refers to the total number of reports received by the regional health bureau over a specified period of time, divided by the total number of reports expected from the health facilities during same period. In order to assess whether the survey was done in a representative year, we extracted the total annual malaria cases recorded in the IDS for Oromia and SNNPR regions over the period July 2004 through June 2007. Annual numbers of cases have been converted to incidence per 1000 using the 2006 population figures.

**Figure 3:**
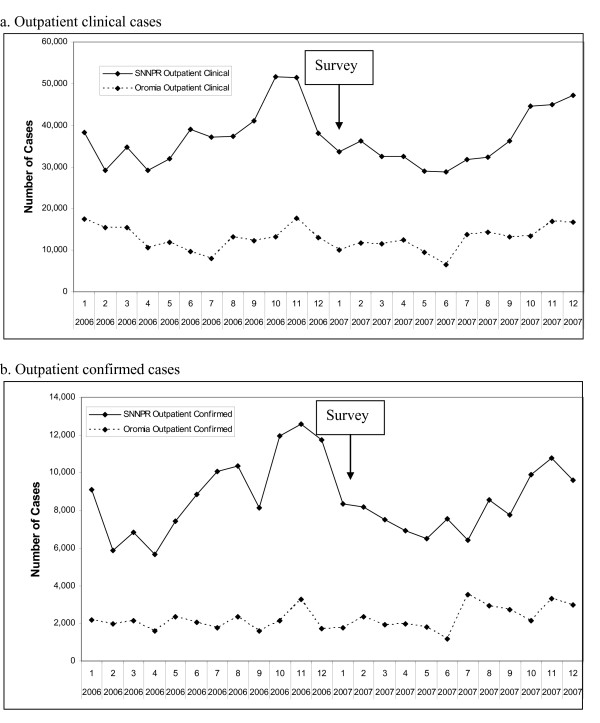
**Number of malaria cases by month reported from health centres and hospitals in Oromia and SNNPR, Jan 2006 to Dec 2007.** a. Outpatient clinical cases. b. Outpatient confirmed cases.

The timing of the survey relative to the seasonal pattern of transmission in the IDS data is shown in Fig 3. The survey in January 2007 was slightly after the peak (in late 2006) of both outpatient clinical malaria cases (Fig 3a) and outpatient confirmed malaria cases (Fig 3b). However, seasonal variation shows no more than a 2 fold difference between the lowest and highest monthly incidence of reported clinical cases in both regions.

The incidence of reported malaria cases in the year prior to the survey (July 2005-June 2006) was ~4 fold lower in Oromia (8.0/1000/year) than in SNNPR (32.3/1000/year). 'Reported malaria cases' means a combination of fever cases that were not tested, and cases which were confirmed positive for malaria. The incidence of confirmed cases alone was lower, and showed a similar ratio between the regions: 1.6/1000/year in Oromia and 8.2/1000/year in SNNPRR. Among confirmed cases, the ratios of P.falciparum to P. vivax differed between the regions and the indicators, being 1.4 among cases recorded in IDS in Oromia, 4.3 for prevalent infection measured in the survey in Oromia, and 2.7 in IDS and 2.1 for prevalence in the survey in SNNPR.

**Figure 4:**
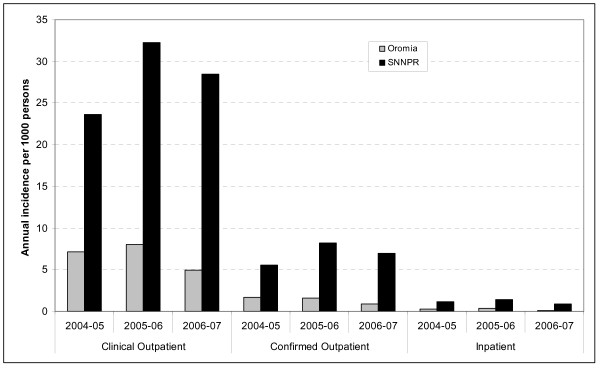
Annual incidence per 1000 persons of outpatient clinical and confirmed malaria cases and inpatient cases, reported from health centres and hospitals, Oromia and SNNPR, July 2004 to June 2007.

To assess annual incidence in July 2006-June 2007 compared to previous years and between regions, the annual incidence of clinical and confirmed malaria cases in both regions are shown in Figure 4 in three categories: outpatient clinical cases, outpatient confirmed cases and inpatient cases. Figure 4 shows that the year including the survey (2006–2007) was not an abnormally low or high malaria year in either region.

## Discussion

Malaria continues to occur in many parts of Oromia and SNNPR regions of Ethiopia, and data from our 2007 survey and from the routine surveillance show that malaria is more prevalent in SNNPR than in Oromia. The incidence of reported clinical malaria cases in the year prior to the survey was four times higher in SNNPR than in Oromia, and the parasite prevalence in our survey was six times higher in SNNPR. In both regions, however, the survey results may be an underestimate of the peak prevalence because the survey was done in January, after the high transmission season that usually goes from August through December. The routine surveillance data also indicate however that, in both regions, malaria incidence in the lowest transmission season is about half of that in peak transmission season. There were some differences in parasite species ratio between the survey and the routine data: less P.falciparum was detected in Oromia than expected from surveillance data. This may represent seasonal variation since the survey was done at one point in time rather than over a full year as in the surveillance data. Apart from seasonal variation, these differences may also relate to differences between falciparum and vivax episodes in treatment seeking (e.g. if a larger proportion of falciparum cases report to health facilities than vivax cases, because of more severe symptoms), or in the duration of infection – which will affect parasite prevalence in the survey but not the incidence in the routine system.

The results of our survey are consistent with findings of other studies conducted within the country, although none are representative surveys of malaria parasite prevalence in the Oromia and SNNPR regions. A parasite prevalence survey in three zones near Lake Ziwai in Oromia from July to September 1994 found an average of 6.8% of slides positive over the period, with 4.8% in December only (P.falciparum 4.5% and P.vivax 2.1%) [15]. The malaria prevalence in pregnant women in three sites of unstable transmission, one of which was Jimma town in Oromia, in surveys in December 2000/January 2001 was 1.8% (70% P.falciparum) [16].

Coverage of key vector control interventions has shown remarkable progress compared to estimates generated by the Ethiopia Demographic and Health Survey (DHS) conducted in 2005 [17]. The Ethiopia DHS did not specifically report on LLINs, which were not commonly available at the time. Therefore we focus the comparison on ITN coverage and use between our survey and the DHS on the use of conventional insecticide treated nets (ITNs), using the DHS definition of either a LLIN or a conventional ITN which had been treated within the last year. In 2005, the proportion of households with at least one net (any type) in Oromia and SNNPR was only 2.8% and 8.2%, respectively, and only 1.9% and 6.6% of households owned at least one ITN. In contrast, in our survey nearly half of the households in each region owned at least one mosquito net and more than one-third of the households owned at least one LLIN. The mean number of ITN/LLIN per household increased from nearly zero in 2005 to 0.5 at the beginning of 2007.

The proportion of people who slept under an ITN/LLIN exhibited a ten-fold increase between 2005 and 2007 (below 3.5% vs. 35.4%). The improvement in net coverage over the period is mainly due to large numbers of nets supplied by the Global Fund for AIDS, TB and Malaria in rounds 2 and 5, and additional net donations plus assistance in procurement and delivery from UNICEF and other donors. For example, 2 million nets were distributed in 2005 alone, and the Global Fund singled out Ethiopia in its 2006 annual report as a country making major strides in scaling up net coverage [18]. Other studies in the Horn of Africa have documented that bed nets confer high protection against malaria infection under field conditions [19]. Assessment of the protective effect of LLIN against malaria in this study relative to other protective and risk factors (including rainfall) are being analyzed separately and will be reported elsewhere.

Similar good news was found for household insecticide spraying. DHS 2005 estimated that 8.5% and 9.1% of households were ever sprayed with insecticide in Oromia and SNNPR, respectively, and only 2.1% of households were sprayed with insecticide in both regions 0–6 months preceding the 2005 DHS [17]. The survey reported here shows that almost one in five households had indoor residual house spraying in the 12 months preceding January 2007.

The current survey was conducted prior to additional planned further mass distribution of LLINs. The baseline results will help to inform the existing gap and gauge progress in LLIN coverage and utilization towards the stated goal of providing a mean of two LLIN per household. The baseline LLIN coverage in these two regions might serve as a reflection of the overall net coverage in the country though regional variations cannot be ruled out. A similar study conducted in Amhara region in December 2006 reported a slightly lower ITN/LLIN coverage [7].

However, LLIN distribution does not necessary translate into LLIN use, and we also designed this survey with the intent of examining if the onchocerciasis program can provide assistance to the malaria program through promotion of LLIN use during annual Mectizan^® ^distribution activities. Currently the onchocerciasis CDDs are being trained to integrate education about LLIN into their 2008 activities. Our hypothesis is that the CDDs will serve as an 'adjuvant' to the Health Extension Workers (HEWs) who will play a pivotal role throughout Ethiopia in the grass roots implementation of the malaria control program. Such a finding would be further evidence that integration of malaria programs with neglected tropical disease programs (NTDs) is good policy [20,21].

The results of this survey compare favourably with that of the routine surveillance and indicate that SNNPR (particularly the eastern and north-eastern areas of the region) bears a higher burden of malaria than Oromia. Future malaria control interventions need to take this into consideration.

## Conclusion

Coverage of key malaria control interventions showed a remarkable increase; household net ownership exhibited nearly ten-fold increase compared to the results of Demographic and Health Survey 2005 when fewer than 5% of households in these two regions owned any nets. The results of the survey as well as the routine surveillance data demonstrated that malaria continues to be a significant public health challenge in these regions–and more prevalent in SNNPR than in Oromia.

## Abbreviations

CDD: Community drug distributors; CDTI: Community-directed treatment with ivermectin; IDS: Integrated disease surveillance; ITN: Insecticide treated net; LLIN: Long-lasting insecticidal nets; RDT: Rapid diagnostic test.

## Competing interests

The authors declare that they have no competing interests.

## Authors' contributions

EBS, TG, PMG, PME, YE, AWM, and FOR designed the survey; EBS, YE, TG, GY, AT, AW, DO and AWM supervised and conducted field work; TE and EBS supervised and conducted microscopy; JN, PMG and YE supervised data management, cleaning, and production of the analysis data set; JN and PMG conducted the analysis; EBS, PMG and JN drafted the manuscript which all authors edited and approved.

## Pre-publication history

The pre-publication history for this paper can be accessed here:


